# Australian Consumers’ Preferences for Food Attributes: A Latent Profile Analysis

**DOI:** 10.3390/foods10010056

**Published:** 2020-12-28

**Authors:** Airong Zhang, Emma Jakku

**Affiliations:** 1 Health & Biosecurity, CSIRO, Brisbane 4102, Australia; 2 Land and Water, CSIRO, Brisbane 4102, Australia; Emma.jakku@csiro.au

**Keywords:** consumer preference, food attributes, latent profile analysis, segmentation

## Abstract

Understanding consumer food preferences can provide agribusinesses with a competitive advantage through meeting consumers’ needs. Consumers’ preferences for food attributes have been extensively examined, focusing on specific aspects of attributes with specific food products. It is less clear how consumers evaluate the relative importance of the key food attributes in general. Applying the commonly adopted classification of food attributes into endogenous attributes (i.e., safety and freshness) and exogenous attributes (i.e., genetically modified (GM)-free and organic), the relative importance of these attributes for consumers was investigated. Furthermore, the heterogeneity of preferences was explored to identify distinct subgroups of consumers who may differ in valuing various food attributes. An online survey of 489 city dwellers in Australia revealed that the endogenous attributes were regarded as the most important in an order of safety and freshness. The exogenous attributes were rated as much less important. Three profiles with distinctive preferences for food attributes were identified: Not Fussy (12% of participants), Quality First (49%) and Choosy (39%). The findings suggest that consumers value the importance of various food attributes in a hierarchical order, and there is significant heterogeneity in consumers’ food preference. The implications of these findings are discussed in the context of food policy and agribusiness decision-making.

## 1. Introduction

Consumers’ decision-making processes around food are complex, dynamic and multidimensional. Recent trends in consumer attitudes and behaviour include an increased focus on food safety and health, partly as a result of high-profile food crises and alarms, new information about diet and health as well as consumer concerns about the application of new technologies in the food sector [1,2,3,4,5]. In addition, traditional purchasing criteria such as price and taste continue to be important [6,7,8]. Understanding consumers’ beliefs and perceptions about the relative importance of different food attributes is important, in part because this is what ultimately influences their purchasing decisions [9,10,11,12]. A better understanding of consumer preferences can help food producers and processors identify nuanced market segments and develop effective and targeted marketing campaigns [6,13,14].

Studies in consumer preferences of food attributes have, so far, focused primarily on two or three particular food attributes in isolation of other attributes, for example, preference for local and organic attributes [15,16], price, taste, environmental friendliness and healthfulness [6,17]; environmental sustainability and food safety [18]; genetically modified (GM) foods and conventional foods [19,20]; food safety [21]; and organic foods, quality, and price [22]. In addition, these attitudes have been examined in relation to specific food products.

Despite the valuable insights these researches have provided, they shed little light on how consumers evaluate those various food attributes comparatively, given that they are often present together in food products. Hence, there is a need to develop understanding of the relative importance of key food attributes. More importantly, certain food attributes may be important for some consumers but not for others. For example, the consumers of organic foods have been characterised as a small core of big spenders [23]. Hence, the examination of preferences for various attributes among different sociodemographics will offer more nuanced understanding of consumer attitudes.

### 1.1. Key Food Attributes

Various food attributes have been explored in relation to consumers’ evaluation of food products. Among the attributes examined, safety, freshness, organic, and GM-free have been applied to a wide range of food products. Hence, they appear to be the common attributes of food products in general.

Food safety has become a predominate concern worldwide due to recurring food scandals and incidents over the past two decades [2,24,25,26,27,28]. The World Health Organisation [29] has identified over 200 foodborne diseases ranging from infectious illness to cancers [30]. In Australia, 707 food recalls were officially recorded between 2010 and 2019 due to safety concerns [31]. Some of these incidents led to health scares. For example, the Salmonella outbreaks in salad led to about 300 people falling ill in February 2016 [32]. These incidents have caused huge public concerns and have led consumers to be increasingly critical about food safety. Freshness is linked to how much a product changes its quality after harvest or production. It has been widely studied and has been identified as a key sensory property of food products, driving preferences [33,34,35,36].

Being increasingly aware of the health, social and environmental consequences of food choices, consumers have become more particular about the processes used to produce their food. Credence attributes such as organic, environmentally friendly and ethical have become important [37,38,39]. These attributes are increasingly used as areas of differentiation in food marketing [38]. Apart from the perceived benefits associated with food safety and freshness, consumers also believe that these attributes offer a positive externality to society, such as creating less damage to environment. Hence, it has been argued that the preferences for food with those attributes are mainly motivated by altruistic values and beliefs [15,38].

Organic food products have become an influential factor in consumers’ food purchase decisions underpinned by increasingly important personal and societal lifestyle values [40,41,42,43]. They are different from conventional foods in their production methods, which are characterised as environmentally and animal friendly, as well as free from conventional pesticides and herbicides [42]. Organic foods are often perceived to be associated with better quality, nutritional value, food safety and taste [42,44,45,46,47]. There is evidence suggesting that consumers are even willing to pay more for organic food products than for conventional products, but with variations among consumer groups (for review, see [48]).

Whereas organic food products have become increasingly popular with consumers over the past decade [44], genetically modified (GM) foods have received mixed reactions, with scepticism and rejection dominating the literature [20,49,50,51,52]. Much of the rejection is caused by uncertainty and perceived risks, as well as environmental-, ethical- and value-related concerns such as being “unnatural” and producers “playing God” [53,54,55,56]. As a result, the benefits associated with GM are discounted [20,57]. A meta-analysis of GM food valuation studies has suggested that, in general, consumers are willing to pay more for GM-free food [53,58].

### 1.2. Categorisation of Food Attributes

It has been argued that food attributes are different in nature and should be distinguished when examining consumer preferences [24]. Various attempts have been made to classify food attributes. The most adopted criterion is to categorise food attributes according to whether the attribute is belief- and value-laden (hence, is exogenous in nature), or centred on the food product itself (hence, is endogenous in nature) [22,24].

Based on the above principles of classifying food attributes, we can argue that the attributes of organic and GM-free are belief- and value-laden and are thus are exogenous to food products. For example, both organic and GM-free have been considered under the heading of ethical preferences [18,38,44,59]. On the other hand, the attributes of safety and freshness are mainly centred on the food product itself. Hence, these attributes are endogenous to the food product itself. Consequently, for the present study, we distinguish these two types of attributes and refer to them as exogenous attributes and endogenous attributes, respectively.

### 1.3. The Present Study

The present study aimed to assess how consumers value the relative importance of various endogenous and exogenous attributes, as well as to identify if there are distinct subgroups among consumers who may differ in what food attributes are important to them. We particularly focused on safety and freshness as representative endogenous attributes, and organic and GM-free as representative exogenous attributes. These attributes were chosen because, as discussed above, they have been identified as influential factors driving consumers’ preference and purchase behaviour and they are common attributes applicable to a wide range of food products.

For the present study, we also included affordable price as a key attribute. This is because the price of food products often becomes an influential product attribute affecting consumer preferences and choice [22,60]. Price is regarded as a cost for a transaction, which can become a barrier for purchasing certain products such as organic foods. Inclusion of price will provide the context for understanding consumer preferences of various attributes from the perspective of affordability.

## 2. Method

### 2.1. Participants and Procedure

A professional research survey company was engaged to recruit participants from the 3 largest cities in Australia (Brisbane, Melbourne and Sydney) and to conduct the online survey between June and July 2016. Participants were paid a small fee by the survey company for their participation. The survey was introduced as an effort to understand consumers’ attitude toward food attributes and related issues in an email invitation with a link to the online survey. In total, 489 people completed the survey. The demographic information of the participants is presented in Table 1. The average household size was 2.76 (*SD* = 1.31).

### 2.2. Measures

The measures included in this paper are part of a larger questionnaire. To measure how important the focal food attributes are to participants, they were asked to think about the food they ate and indicate how important each attribute was to them on a 5-point scale (1 = not important at all, 5 = extremely important). The food attributes included safety, freshness, organic, GM-free and affordable price.

### 2.3. Data Analysis

To examine the relative importance of the attributes, a series of paired-sample T-tests was conducted. To examine the heterogeneity of the attribute preferences among consumers, latent profile analysis (LPA) was applied. Latent profile analysis aims to categorise people into meaningful groups that are similar in their responses to observed indicators [61]. A series of 4 LPAs were conducted to identify similar patterns in demographics and preference of food attributes using Mplus Version 7.4 [62]. In measuring model fit, we examined 4 commonly used fit statistics. Akaike information criterion (AIC) and sample-size-adjusted Bayesian information criterion (ssaBIC) were used to assess model fit, with lower values indicating better fit. Lo-Mendell-Rubin adjusted LRT (LMR-ALRT) test was applied to examine whether the k profile model was better than the k −1 profile model, with *p* > 0.05 for the k profile model indicating that the later model should be rejected in favour of the former. Entropy provides an index of profile classification accuracy and has a value range of 0.0 to 1.0, with higher values indicating better differentiation of individuals into profiles. Values greater than 0.80 are considered to have good classification quality [63]. Further, to examine the differences in demographics and food attributes between the groups identified through the latent profile analysis, a series of analysis of variance (ANOVA) was conducted.

## 3. Results

### 3.1. Relative Importance of Food Attributes

**Figure 1:**
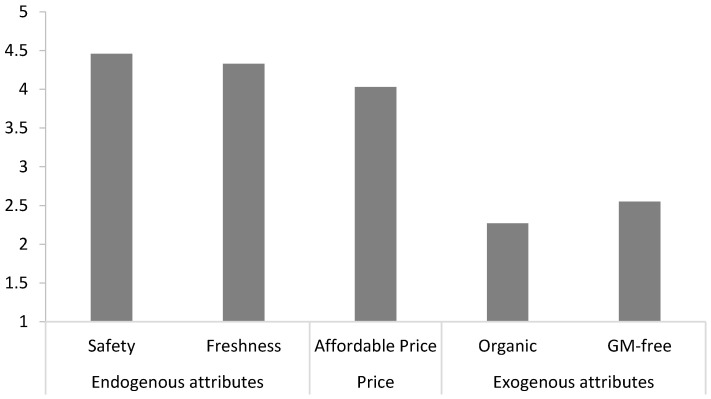
Reported importance of food attributes (1 = not important at all, 5 = extremely important).

Figure 1 presents the means of reported importance for the measured food attributes. Both endogenous attributes were rated quite high. Food safety (*M* = 4.46, *SD* = 0.77) was the most important attribute, closely followed by freshness (*M* = 4.33, *SD* = 0.76). The exogenous attributes organic (*M* = 2.27, *SD* = 1.18) and GM-free (*M* = 2.55, *SD* = 1.37) were rated as much less important, while the importance of affordable price (*M* = 4.03, *SD* = 0.84) was rated high as well. The paired-sample T-tests analyses indicated that the levels of importance attached to each of the attributes were all significantly different from each other (*p* ≤ 0.001).

### 3.2. Latent Profile Analysis

The model fit indices from the latent profile analysis are presented in Table 2. The LMR-ALRT test indicated that the four-profile model was significantly worse than the three-profile model, with *p* = 0.206. In addition, the values of AIC and ssaBIC for the three-profile model were lower than the two-profile model, and the Entropy value for the three-profile was high (0.859). Hence, the three-profile solution was selected as optimal to describe food attribute preference.

Table 3 presents the demographic characteristics for the three profiles and summarises the within-profile item means and standard errors. To reflect the characteristics of the three profiles, they are labelled as Not Fussy, Quality First and Choosy.

The Not Fussy group (12% of participants) was dominated by individuals who tended to be middle-aged males (67% males) with average education levels. The Quality First group (49% of participants) mainly comprised of individuals who tended to be middle-aged, much like the Not Fussy groups was, but with more females (55% females) and lower education levels. The Choosy group (39% of participants) included individuals who were comparatively younger, more females (59% females) and the most educated.

In responding to the endogenous food attributes, it appears that participants from the Quality First and Choosy groups displayed a similar pattern by regarding food safety and freshness as very important, while those from the Not Fussy group scored the importance of these attributes significantly less comparatively. In particular, participants from the Quality First and Choosy groups equally rated food safety highly important, and those from the Not Fussy group regarded food safety as moderately important. In relation to freshness, the Choosy group rated it as very important, closely followed by the Quality First. The Not Fussy group was the lowest but still regarded freshness reasonably important.

In relation to the exogenous food attributes of organic and GM-free, it appears that participants from the Choosy group attached the highest importance to these attributes, while the participants from the Quality First and Not Fussy groups regarded these attributes were not that important at all.

Affordable price was considered to be important by all three groups, but with significant differences between them. Interestingly, although the Choosy group valued the importance of both endogenous and exogenous attributes highly, they were the most cost-cautious consumers, indicating that affordable price was very important for them. Comparatively, the level of importance rated by the Not Fussy group was the lowest.

## 4. Discussion

The present study examined how urban consumers evaluate the importance of various endogenous and exogenous food attributes. Moreover, the heterogeneity in consumer preferences for those attributes was investigated using latent profile analysis to provide more nuanced understanding of market segments, which can help food producers and processers develop effective and targeted marketing campaigns as well as creating opportunities for value-added and differentiated products.

The results showed that consumers valued the traditional traits (i.e., endogenous attributes), with safety and freshness as the most important food attributes. In particular, food safety was non-negotiable and the most important attribute. It is possible that the highly publicised food safety incidents, as well as debates over pesticide and herbicide usage in farming, may have resulted in increased risk perceptions, concerns and anxiety, despite the fact that food production has never been safer and better regulated and controlled. This finding has great implication for the food industry, policymakers and regulatory bodies with respect to risk management, risk assessment, and risk communication to build consumer confidence in food safety. Although the exogenous attributes including organic and GM-free have become more prominent and have appeared to be a trend in food markets over the past decade [18], our findings suggest that consumers regarded them as much less important in comparison to the endogenous attributes.

The latent profile analysis provides a nuanced understanding of attribute preferences, showing that consumers are individuals with highly variable preferences for various food attributes. The LPA analysis revealed three distinctive profiles in food preferences (see Table 3). The Not Fussy consumers (who were more likely middle-aged males with average education) showed a consistent pattern of attaching the lowest levels of importance to all food attributes. On the other hand, the Choosy consumers (who were more likely younger female with higher education) attached very high importance to safety and freshness, high importance to GM-free and moderate importance to organic. This finding is in line with the findings of previous research showing that the exogenous attributes appeal to a small core of big spenders [23]. The Quality First consumers (who were more likely middle-aged females with the lowest levels of education) emphasised food safety and freshness as very important as the Choosy did. However, they displayed lower levels of importance rating on GM-free and organic, similar to the Not Fussy group.

The present study highlights the importance of examining consumers’ food preferences where key attributes are considered. For example, GM foods have been cast in the light of public opposition due to concerns over risks, ethics and value (for reviews, see [54,64,65]). It is likely that these studies have largely focused on examining consumers’ perceptions of and attitudes toward GM foods in isolation of other food attributes. In contrast, when investigating consumers’ attitudes toward GM foods in combination with other food attributes, our results suggest that GM-free was one of the least important attributes for consumers. It was only the highly educated younger consumers (39% of participants) who emphasized the GM-free attribute. Even so, GM-free was still perceived as less important compared to the endogenous attributes. For most participants (61%), GM-free was only rated at about “a little bit important.” The finding of preferences for GM-free food by the highly educated younger consumers has implications for policymakers and food producers. So far, consumers’ poor scientific knowledge and understanding have been regarded as contributing to the public’s opposition to GM foods. Consequently, communicating science and educating consumers have been suggested as essential for public acceptance of GM technology in food production [66]. Our findings suggest that higher education levels do not necessarily translate to greater levels of acceptance of GM food products. However, it is important to note that the present study did not account for the differences between general educational attainment and specific knowledge about GM in food production.

Furthermore, our findings suggest that affordable price may be a key constraint for marketing food products which possess exogenous attributes. Affordable price was regarded as quite important by all participants, especially for the Choosy consumers. This indicates that, although the exogenous attributes were important for them, there may be price constraints for those consumers to turn their preference into purchasing behaviour. This finding is consistent with early research suggesting that consumers prefer organic food products but the price difference between conventional and organic food products needed to be minimal [15].

Whereas the findings of the present study provide insights into what food attributes matter to Australian urban consumers, caution needs to be taken when applying the findings. Consumers’ food preference has been shown to be influenced by culture [67], geographic location (e.g., rural vs. urban) [28,68] and wealth [69,70,71]. Future research should investigate how consumers evaluate the relative importance of the key food attributes across cultures and from different economic developmental stages. Such understanding will enable food producers and processers to meet consumers’ specific needs in different cultural and economic development settings. In addition, the goal of the present study was to understand how consumers evaluate the relative importance of key food attributes in relation to their daily food. As such, the findings may not apply to food service settings such as restaurants.

## 5. Conclusions

In conclusion, our results indicate that, although exogenous food attributes including organic and GM-free have become popular and have taken up a noteworthy market share over the past decade, the traditional food attributes such as safety and freshness are still the first and foremost traits for Australian consumers. More importantly, consumers value the importance of various food attributes in a hierarchical order, and there is significant heterogeneity in consumers’ food preference patterns. These findings have significant implications for decision-making among policymakers, producers and agribusinesses. Specifically, food safety is regarded as non-negotiable and as the most important attribute by all participants, indicating that it is crucial to build consumer confidence in food safety through risk management, risk assessment and risk communication. Moreover, although the exogenous attributes including organic and GM-free have appeared to be trend in food markets over the past decade [18], our findings suggest that consumers regarded them as much less important in comparison to the endogenous attributes and that they only appealed to particular consumer segments. Paradoxically, our findings show that consumers with higher level educations appear more likely to oppose GM food. This suggests that the current strategy of communicating science and educating consumers for the public acceptance of GM technology in food production may not be effective. Genetic intervention for food production provides important opportunities to combat the impacts of climate change and ensure food security. Future research should explore the types of effective communication and public participation strategies necessary to achieve consumers’ acceptance. This study highlights the inherent complexity of communicating a diverse range of food attributes to consumers, given the differences in preferences that exist.

## Figures and Tables

**Table 1:** Demographic characteristics.

	*N*	%
*Gender*		
Male	227	46.4%
Female	262	53.6%
*Age*		
18–24 years	43	8.8%
25–34 years	82	16.8%
35–44 years	85	17.4%
45–54 years	88	18.0%
55–64 years	97	19.8%
65 or older	94	19.2%
*Education*		
Did not complete Year 12	49	10.0%
Completed Year 12	88	18.0%
Postsecondary qualification	106	21.7%
Undergraduate degree	146	29.9%
Postgraduate degree	100	20.4%
*Household income*		
Under AUD 30,000	60	12.3%
AUD 30,001–60,000	129	26.4%
AUD 60,001–80,000	92	18.8%
AUD 80,001–100,000	74	15.1%
AUD 100,001 or more	134	27.4%

**Table 2:** Latent profile fit statistics for attribute preference model with one to four profiles.

No. of Profiles	AIC	ssaBIC	LMR-ALRT	LMR-ALRT *p*-Value	Percentage in Each Profile				
					Entropy	Prof. 1	Prof. 2	Prof. 3	Prof. 4
1	14,276	14,360	−	−	−	100	−	−	−
2	13,898	13,929	394	<0.001	0.870	57	43	−	−
3	13,748	13,790	170	0.006	0.859	14	48	38	−
4	13,686	13,908	82	0.206	0.787	13	29	21	37

**Table 3:** Demographic characteristics, means and standard errors of food attributes for the three profiles.

	Not Fussy (*n* = 61)	Quality First (*n* = 239)	Choosy (*n* = 189)	Chi-Square Test
	%	%	%	
*Age*				
18–24 years	4.7	46.5	48.8	*X*^2^ (10, *N* = 489) = 19.45, *p* = 0.035
25–34 years	9.8	50.0	40.2	
35–44 years	14.1	34.1	51.8	
45–54 years	13.6	48.9	37.5	
55–64 years	17.5	52.6	29.9	
65 years or older	10.6	58.5	30.9	
*Gender*				
male	18.1	47.6	34.4	*X*^2^ (2, *N* = 489) = 12.88, *p* = 0.002
female	7.6	50.0	42.4	
*Education*				
did not complete Year 12	14.3	57.1	28.6	*X*^2^ (8, *N* = 489) = 8.74, *p* = 0.364
completed Year 12	9.1	58.0	33.0	
postsecondary qualification	11.3	50.9	37.7	
undergraduate degree	13.7	42.5	43.8	
postgraduate degree	14.0	44.0	42.0	
*Income per annum*				
under AUD 30,000	13.3	48.3	38.3	*X*^2^ (8, *N* = 489) = 5.60, *p* = 0.692
AUD 30,001–$60,000	10.9	49.6	39.5	
AUD 60,001–$80,000	9.8	47.8	42.4	
AUD 80,001–$100,000	17.6	54.1	28.4	
AUD 100,001 or more	12.7	46.3	41.0	
	**Not Fussy** **(*n* = 61)**	**Quality First** **(*n* = 239)**	**Choosy** **(*n* = 189)**	**ANOVA**
	***M (SE)***	***M (SE)***	***M (SE)***	
*Household size*	2.61 (0.17)	2.67 (0.09)	2.93 (0.11)	*F* (2, 486) = 2.59, *p* = 0.076
*Endogenous attributes*				
Safety	2.98 (0.17)	4.71 (0.04)	4.67 (0.05)	*F* (2, 486) = 382.65, *p* < 0.001
Freshness	3.58 (0.11)	4.31 (0.07)	4.61 (0.05)	*F* (2, 486) = 52.71, *p* < 0.001
*Exogenous attributes*				
Organic	1.82 (0.17)	1.68 (0.06)	3.17 (0.10)	*F* (2, 486) = 154.08, *p* < 0.001
GM-free	1.91 (0.17)	1.59 (0.06)	3.98 (0.07)	*F* (2, 486) = 607.47, *p* < 0.001
*Affordable price*	3.64 (0.12)	4.01 (0.06)	4.20 (0.06)	*F* (2, 486) = 11.56, *p* < 0.001

Note: All food attributes were rated on a 5-point scale (1 = not important, 5 = extremely important).

## Data Availability

Data available on request due to privacy or ethical requirement.
